# Hearing Loss and Health Care Seeking

**DOI:** 10.1093/geroni/igab046.2343

**Published:** 2021-12-17

**Authors:** Nicholas Reed

**Affiliations:** Johns Hopkins Bloomberg School of Public Health, Baltimore, Maryland, United States

## Abstract

Hearing loss is common among older adults. Hearing loss is associated with increased health care expenditures, risk of 30-day readmission, and longer length of hospital stay. However, little is known about behaviors and attitudes in seeking care. In this cross-sectional analysis, we examined data from the 2016 Medicare Current Beneficiary Survey (MCBS) datasets. Participants are asked to describe their self-perceived trouble hearing. Health care seeking attitudes were assessed on all study participants in 2016 via self-report avoidance or delay of care, personal health concerns, and sharing health status. Multivariate regression models adjusted for demographic/socioeconomic characteristics and general health determinants were used to explore the association between trouble hearing and outcomes. In the 2016 MCBS, 12,140 Medicare beneficiaries, representing 51 million with survey weights, answered questions on help-seeking attitudes. In the sample, 55.6% reported no trouble hearing, while 38.8% and 5.5% reported a little trouble and a lot of trouble hearing, respectively. Those with a lot of trouble hearing were more likely to report avoiding doctors (Odds Ratio [OR] = 1.35; 95% Confidence Interval [CI] = 1.09 – 1.67) and delaying care (OR = 1.47; 95% CI = 1.19 – 1.82). However, no differences were found in personal health concerns or willingness to share health status with others. Poorer health care seeking behaviors may help explain higher costs associated with hearing loss as avoidance of care can exacerbate health problems. Further work is needed to understand underlying reasons and whether addressing hearing loss modifies the observed association.

